# Formulation and optimization of itraconazole polymeric lipid hybrid nanoparticles (Lipomer) using box behnken design

**DOI:** 10.1186/s40199-014-0087-0

**Published:** 2015-01-21

**Authors:** Balaram Gajra, Chintan Dalwadi, Ravi Patel

**Affiliations:** Department of Pharmaceutics & Pharmaceutical Technology, Ramanbhai Patel College of Pharmacy, Charotar University of Science and Technology, CHARUSAT Campus, Changa, 388 421 Gujarat India; Department of Pharmaceutics, Indian Institute of Technology, Banaras Hindu University (IIT-BHU), Varanasi, 221 005 UP India

**Keywords:** Polymeric lipid hybrid nanoparticles, Box-behnken design, Entrapment efficiency, Drug loading, Optimization

## Abstract

**Background:**

The objective of the study was to formulate and to investigate the combined influence of 3 independent variables in the optimization of Polymeric lipid hybrid nanoparticles (PLHNs) (Lipomer) containing hydrophobic antifungal drug Itraconazole and to improve intestinal permeability.

**Method:**

The Polymeric lipid hybrid nanoparticle formulation was prepared by the emulsification solvent evaporation method and 3 factor 3 level Box Behnken statistical design was used to optimize and derive a second order polynomial equation and construct contour plots to predict responses. Biodegradable Polycaprolactone, soya lecithin and Poly vinyl alcohol were used to prepare PLHNs. The independent variables selected were lipid to polymer ratio (X_1_) Concentration of surfactant (X_2_) Concentration of the drug (X_3_).

**Result:**

The Box-Behnken design demonstrated the role of the derived equation and contour plots in predicting the values of dependent variables for the preparation and optimization of Itraconazole PLHNs. Itraconazole PLHNs revealed nano size (210 ± 1.8 nm) with an entrapment efficiency of 83 ± 0.6% and negative zeta potential of −11.7 mV and also enhance the permeability of itraconazole as the permeability coefficient (P_app_) and the absorption enhancement ratio was higher.

**Conclusion:**

The tunable particle size, surface charge, and favourable encapsulation efficiency with a sustained drug release profile of PLHNs suggesting that it could be promising system envisioned to increase the bioavailability by improving intestinal permeability through lymphatic uptake, M cell of payer’s patch or paracellular pathway which was proven by confocal microscopy.

## Background

The frequency of acquiring bacterial, viral, or fungal infectious diseases increase each year due to the ease of transmission from person to person. From many forms of the infection, invasive fungal infections have become more common in recent years, with a nearly 500% growth in the incidence of blood stream infection with Candida spp. since the 1980 [1]. The azole antifungal agents represent a major drug class in the treatment of wide variety of fungal infections. These drugs can be divided in two main groups: the imidazoles and the triazoles [2].

Itraconazole (ITZ) is a potent triazole antifungal with broad spectrum of activity against fungal species and more efficacious for the treatment of both systemic and superficial fungal infections [3]. ITZ is widely clinically used for a variety of serious fungal infections in normal and immunocompromised hosts, including *Aspergillosis*, *Cryptococcus*, *Candida*, *Blastomyces*, disseminated *Penicillium mameffei* infections and *Histoplasma capsulatum var. capsulatum* and also it has less nephrotoxicity than Amphotericin B [4].

One of the problem with ITZ is its highly hydrophobic characteristics and extremely weak basicity with aqueous solubility of approximately 1 ng/ml at neutral pH [2]. The Sporanox® marketed oral capsule and solution formulation of the ITZ are not allowed to be used in patients with impaired renal function and aged person. It is not because of the toxicity of the drug itself, but the adjuvant hydroxypropyl-β-cyclodextrin (HP-β-CD). Each milliliter of Sporanox® solution and capsule contains 10 mg of ITZ solubilised by 400 mg of HP-β-CD as an inclusion complex. Following a single intravenous dose of 200 mg Sporanox® to the subjects with severe renal impairment, clearance of HP-β-CD was 6-fold reduced compared with subjects with normal renal function [3]. Hence, a development of oral formulation of ITZ without HP-β-CD is very much important.

The classical polymer lipid hybrid nanoparticles (PLHN) are composed of liposomes and polymeric nanoparticles into a single delivery system. This type of nanoparticles are typically comprised of two distinct functional components: (i) a hydrophobic or hydrophilic polymeric core where poorly water-soluble or highly water soluble drugs are incorporated with high loading yields; (ii) a lipid layer surrounding the core that acts as a highly biocompatible shell and as a molecular fence to promote drug retention inside the polymeric core [5].

There are several pathways used by molecules to cross the epithelial cell barrier, which include transcellular (transport through the cell, with crossing of the cell membranes), paracellular (transport between adjacent cells), and transcytosis through enterocytes. Transcellular pathways through M cells is one of the mechanisms to transport nanoparticles across the intestinal barrier. M cells are associated with Peyer’s Patches (PP), an organized component of the gut-associated lymphoid tissue (GALT) [6]. M cells have several properties that allow for adherence by NPs, such as reduced proteases, lack of mucus secretion, and a sparse glycocalyx [7]. A number of approaches have been used to target nanoparticles to M cells. Various nanoparticles like chitosan nanoparticle [8,9], solid lipid nanoparticle [10], polymeric nanoparticle [11-13] and nanoemulsion [14] are capable of enhancing intestinal absorption of poorly water soluble and permeable drugs.

The distinct advantage of this PLHNs have been demonstrated to include the unique advantages of both liposomes and polymeric nanoparticles while excluding some of their intrinsic limitations, thereby holding great promise as a delivery vehicle for various drugs [15]. In the present study emulsification solvent evaporation method was used to prepare PLHN and effect of different independent variables were checked on particle size and entrapment efficiency.

## Material and methods

### Materials

Poly (ɛ-caprolactone) (PCL) (Mw 70,000-90,000) was supplied as a gift sample from Sigma Aldrich, USA. Itraconazole was provided as a gift sample from Intas Biopharmaceutical Ltd, Ahmedabad, India. Soya lecithin 30%, Polyvinyl alcohol and all other Materials like Dichloromethane (DCM), Tetrahydrofuran (THF) and Mannitol (PVA) were purchased from Himedia laboratories Pvt. Ltd, Mumbai, India. Double Distilled Water was used throughout the experiment.

### Preparation of polymer lipid hybrid nanoparticles

PLHNs were prepared by the single emulsification evaporation method. In this method PCL and ITZ were dissolved into the DCM. Soya Lecithin with lipid to polymer ratio of 1:10 was dissolved into the aqueous phase [16]. In order to facilitate the solubilisation of the Soya Lecithin, water miscible organic solvent Tetrahydrofuran (4% v/v) was added into the aqueous solution. Polyvinyl Alcohol (PVA) was added as a stabilising agent (0.5 to 1.5% w/v) into the aqueous phase. The resulting PCL solution was then added into the aqueous solution drop wise with continuous stirring and kept aside for 1 to 2 hr to evaporate the DCM [17]. Then dispersion was centrifuged at 12,000 rpm for 30 min at room temperature and the pellet was redispersed in the double distilled water. The dispersion was sonicated and frozen at −90°C for 3 hr in a deep freezer and freeze dried (Benchtop K freeze dryer, Virtis, 4KBTZL/105, USA).

### Optimization of PLHNs by box-behnken design

A Box-Behnken statistical design with 3 factors, 3 levels, and 15 runs was selected for the optimization study and the Design Expert® 8.0.6 software was used [18]. The independent variables selected were lipid to polymer ratio (X_1_), concentration of surfactant (X_2_) and concentration of drug (X_3_) and dependent variables were particle size (Y_1_) and entrapment efficiency (% EE) (Y_2_) (Table 1) with high, medium and low level. A checkpoint analysis was performed to confirm the role of the derived polynomial equation and contour plots in predicting the responses [19]. Optimization was performed to find out the level of independent variables (X_1_, X_2_, and X_3_) that would yield a minimum value of the particle size (Y_1_) and maximum value of EE (Y_2_).

**Table 1:** **Variables and levels in Box-Behnken design**

		**Level**		
**Independent variables**		**−1**	**0**	**+1**
**X**_**1**_	Lipid to Polymer ratio	1:1	1:5	1:10
**X**_**2**_	Concentration of surfactant (% w/v)	0.5%	1%	1.5%
**X**_**3**_	Concentration of drug (% w/v)	0.06%	0.12%	0.18%
**Dependent variables**				
**Y**_**1**_	Particle Size (nm)			
**Y**_**2**_	% Entrapment Efficiency (nm)			

### Particle size

Particle size was measured by Dynamic light Scattering using the particle size Analyzer (Malvern Zetasizer S90, UK). All measurements were taken by scattering light at 90° and temperature of 25°C. Dispersion was centrifuged at 12,000 rpm for 30 min at room temperature. Supernant was discarded and the resultant pellet was redispersed in double distilled water. Dispersion was then appropriately diluted for the particle size measurement [20].

### % Entrapment efficiency (% EE) and drug loading

Dispersion was centrifuged at 12,000 rpm for 30 min at room temperature, supernant was discarded, the obtained pellet was dissolved in DCM and drug concentration was analysed by UV/Visible Spectrophotometer at 264 nm [21]. Drug loading was determined by the direct method as described for the EE. Measured amount of final freeze dried formulation was dissolved into the DCM and analysed U.V.Visible Spectrophotometer at 264 nm. It was also calculated by the indirect method by the Equation 1.

1 $$ Drug\  Loading\kern0.5em =\kern0.5em \frac{\left[ Amount\  of\ ITZ\  entrapped\right]}{\left[ Amount\  of\ ITZ\  Added+ Amount\  of\  Excipients\  Added\right]} $$

### Fourier transmission infrared spectroscopy (FTIR)

The samples were weighed approximately, homogenously dispersed in dried KBr in a mortar and pestle, and compressed under vacuum with compression force using round flat face punch for three minutes to produce pellet compact. The pellet was placed in the IR light path and the IR spectra were recorded using a FTIR spectrophotometer (NICOLET 6700, Thermo Scientific, USA). Spectrum was recorded in the wavelength region of 4000–400 cm − 1 [20].

### Differential scanning calorimetry (DSC)

DSC Analysis was conducted using the Differential Scanning Calorimeter (DSC-60, Shimadzu, Japan). Sample curves were recorded at a scan rate of 10°C/min from 50 to 300°C. Each powder sample, 5–10 mg was analysed by same procedure. DSC of ITZ, PCL, soya lecithin, Mannitol, PVA, physical mixture and freeze dried final formulation was conducted to show the compatibility of drug with excipients and loading of the drug in to the polymeric matrix [22].

### Powder x-ray diffraction (PXRD)

PXRD of various samples was recorded at room temperature with X-Ray Diffractometer (D2Phaser-brukker, USA). The samples were scanned from the 5° to 50° (2θ) with a step size 0.02° and a step interval of 0.1 Sec [3].

### Transmission electron microscopy (TEM)

TEM of PLHNs was performed following negative staining with Phosphotungstic acid (PTA) [5]. A drop of dispersion (1 mg/ml) was placed on copper grids followed by the addition of a drop of PTA. At the end of 3 min, excess liquid was removed, the grid air-dried and imaging conducted, using a transmission electron microscope (Holland Technai 20, Phillips, Holland) [15].

### Zeta potential

The zeta potential of the dispersion was measured by determining the electrophoretic mobility using the Zetasizer (Malvern Zetasizer ZS90, UK). Dispersion was centrifuged at 12,000 rpm for 30 min at room temperature. Supernant was discarded and the resultant pellet was redispersed in double distilled water using ultrasonic probe system for 1 min with 50 s pulse at 200 v. Dispersion was then appropriately diluted and zeta potential was measured [5].

### In-vitro drug release study

Drug release was performed by dialysis method. Dispersion was filled in dialysis tube (2.4 nm pore size, Himedia, India). Drug release was initiated by immersing the dialysis tube in 200 ml of release media on the magnetic stirrer at 37 ± 5°C and 50 rpm [23]. Various release media were used for the release study like pH 7.4 phosphate buffer, 0.1 N HCL, pH 6.8 phosphate buffer with 3% SLS. Aliquots (5 ml) were withdrawn at specified time points and drug concentration was measured by UV/Visible Spectrophotometer at 264 nm. The release data was fitted with different kinetic models such as zero order, first order, Higuchi and Korsmeyer-Peppas model.

### Ex-vivo permeation study

Male Wistar rats (250–320 gm) were sacrificed by the humane method. Permission for study was obtained from the institutional animal ethics committee (Protocol No. RPCP/IAEC/2011-2012/MPH-PT-13). All the procedures were followed as per guidelines of committee for the purpose of control and supervision of experiment on animals (CPCSEA), Division of Animal Welfare, Ministry of Forests and Environment, Government of India. After rats were sacrificed, the small intestine was immediately excised and placed into ice-cold, bubbled (carbogen, 95:5 O2/CO2) Ringer buffer. The jejunum, 20 cm distal from the pyloric sphincter was used. The tissue was rinsed with ice-cold standard Ringer buffer to remove luminal content and cut into segments. The freeze dried PLHN sample was reconstituted with one ml of phosphate buffer pH 6.8 [24]. Resultant sample was placed in lumen of intestine tied from one side and then tied from other side. The tissue was placed into organ bath filled with 40 mL of phosphate-buffer pH7.4. Continuous aeration and constant temperature of 37 ± 0.5°C were maintained. Samples were taken from the receptor chamber at predetermined time interval and replaced with equal volume of buffer. Aliquots were assayed for the drug content using U.V. Visible Spectrophotometer at 264 nm [25]. It was compared with the simple drug solution in phosphate buffer pH 6.8. Percentage drug permeation and permeability enhancement ratio was calculated from the Equation 2 and 3, respectively [26].

2 $$ Papp=\frac{dQ}{dt}\times \frac{1}{ACo} $$

Where dQ/dt is the steady-state appearance rate on the acceptor side of the tissue, A is the area of the tissue (cm2) and Co is the initial concentration of the drug in the donor Compartment.

3 $$ \mathrm{Permeability}\ \mathrm{Enhancement}\ \mathrm{ratio}\kern0.5em =\kern0.5em \frac{Papp\  of\  the\  nanoparticle\  formulation}{Papp\  of\  the\  drug\  solution} $$

### In-vitro cellular uptake study with confocal laser scanning microscopy (CLSM)

For the cell uptake studies, PLHNs were labelled with fluorescent dye, Rhodamine B and placed into the lumen of the intestine, and kept for 1 hr into the phosphate buffer saline then the tissue was preserved in to the incubation media i.e. 10% formalin for the CLSM study [27]. The block was prepared using cryoprotectant embedding medium. The cross section of the intestinal tissue of 5 μm thickness was taken by cryomicrotome (CM1850, Leica) at −20°C. The section was placed on the slides coated with poly-L-lysine. The slides were incubated at 37°C for the 20 min for the fixation of the section. The slides were examined by CLSM (Zeiss LSM S10 META) through the z axis. Optical excitation was carried out with 480 nm and fluorescence emission was detected above 520 nm for Rhodamine B [28].

### Stability study

For stability study, freeze dried ITZ-PLHNs were stored at room temperature (~25°C), refrigerator (4° to 8°C) and accelerated condition (Temperature: 40 ± 2°C, Relative humidity: 75% ± 5) over a period of 45 days in stopper glass vials. Samples were evaluated for particle size and drug content on 15th, 30th and 45th day. Chemical stability during the storage was checked by FTIR spectrophotometer after 45th day of storage [20].

## Results

### Preparation of PLHN

In the method of preparation, DCM diffuses quickly into the aqueous solution, leaving PCL to precipitate and form nanoparticles. Soya lecithin was self-assemble on the surface of polymer nanoparticles through hydrophobic interactions to reduce the system’s free energy. The hydrophobic tail of lipids was attached to the hydrophobic polymer core and the hydrophilic head group of lipids extend into the external aqueous environment [17].

### Optimization of polymer lipid hybrid nanoparticle by box-behnken design

All the batches of PLHNs were evaluated for the particle size (Y1) and entrapment efficiency (Y2) and the results are shown in the Table 2. Full model polynomial equations for the Particle size and entrapment efficiency are as follows:

**Table 2:** **Box-behnken experimental design with measured responses**

**Batch No**	**X**_**1**_	**X**_**2**_	**X**_**3**_	**Particle size Y**_**1**_ **(nm)**	**Entrapment efficiency Y**_**2**_ **(%)**
**PLN1**	−1	−1	0	251.0 ± 0.7	80.5 ± 0.3
**PLN2**	1	−1	0	353.0 ± 0.2	83.0 ± 0.2
**PLN3**	−1	1	0	214.0 ± 0.03	78.7 ± 0.07
**PLN4**	1	1	0	240.0 ± 1.2	83.0 ± 0.8
**PLN5**	−1	0	−1	244.0 ± 0.67	80.0 ± 0.64
**PLN6**	1	0	−1	248.0 ± 0.8	83.4 ± 0.4
**PLN7**	−1	0	1	234.0 ± 0.9	77.4 ± 0.0.4
**PLN8**	1	0	1	264.0 ± 1.31	81.2 ± 0.09
**PLN9**	0	−1	−1	319.0 ± 0.02	81.8 ± 0.8
**PLN10**	0	1	−1	223.0 ± 0.5	80.1 ± 0.32
**PLN11**	0	−1	1	344.0 ± 0.45	81.0 ± 0.34
**PLN12**	0	1	1	234.0 ± 0.51	78.0 ± 0.23
**PLN13**	0	0	0	245.0 ± 0.32	79.0 ± 1.02
**PLN14**	0	0	0	243.0 ± 0.08	79.9 ± 0.02
**PLN15**	0	0	0	240.0 ± 0.4	80.0 ± 0.05

For Particle Size,

4 $$ \begin{array}{l}{\mathrm{Y}}_1\kern0.5em  = +242.67 + \kern0.5em 13.87{\mathrm{X}}_1\left(\mathrm{P}\kern0.5em =\kern0.5em 0.0057\right)\ \hbox{-} \kern0.5em 50.88{\mathrm{X}}_2\kern0.5em \left(\mathrm{P}\kern0.5em =\kern0.5em 0.0001\right) + \kern0.5em 5.25{\mathrm{X}}_3\kern0.5em \left(\mathrm{P}\kern0.5em =\kern0.5em 0.1403\right)\kern0.5em \hbox{-} \\ {}6.25{\mathrm{X}}_1{\mathrm{X}}_2\left(\mathrm{P}\kern0.5em =\kern0.5em 0.2005\right) + \kern0.5em 6.50{\mathrm{X}}_1{\mathrm{X}}_3\left(\mathrm{P}\kern0.5em =\kern0.5em 0.1858\right)\ \hbox{-} \kern0.5em 3.50{\mathrm{X}}_2{\mathrm{X}}_3\left(\mathrm{P}\kern0.5em =\kern0.5em 0.4466\right)\\ {}+\kern0.5em 1{{.04\mathrm{X}}_1}^2\kern0.5em \left(\mathrm{P}\kern0.5em =\kern0.5em 0.8227\right) + \kern0.5em 33{{.54\mathrm{X}}_2}^2\kern0.5em \left(\mathrm{P}\kern0.5em =\kern0.5em 0.0006\right) + \kern0.5em 3{{.79\mathrm{X}}_3}^2\kern0.5em \left(\mathrm{P}\kern0.5em =\kern0.5em 0.4294\right)\\ {}\left({\mathrm{R}}^2\kern0.5em =\kern0.5em 0.9869\right)\end{array} $$

For % Entrapment Efficiency,

5 $$ \begin{array}{l}{\mathrm{Y}}_2\kern0.5em =\kern0.5em +\kern0.5em 79.63\kern0.5em +\kern0.5em 1.75{\mathrm{X}}_1\kern0.5em \left(\mathrm{P}\kern0.5em =\kern0.5em 0.0006\right)\ \hbox{-} \kern0.5em 0.82{\mathrm{X}}_2\left(\mathrm{P}\kern0.5em =\kern0.5em 0.0160\right)\ \hbox{-} \kern0.5em 0.96{\mathrm{X}}_3\kern0.5em \left(\mathrm{P}\kern0.5em =\kern0.5em 0.0085\right) + \kern0.5em 0.46{\mathrm{X}}_1{\mathrm{X}}_2\\ {}\left(\mathrm{P}\kern0.5em =\kern0.5em 0.2153\right) + \kern0.5em 0.10{\mathrm{X}}_1{\mathrm{X}}_3\kern0.5em \left(\mathrm{P}\kern0.5em =\kern0.5em 0.7647\right)\ \hbox{-} \kern0.5em 0.32{\mathrm{X}}_2{\mathrm{X}}_3\kern0.5em \left(\mathrm{P}\kern0.5em =\kern0.5em 0.3657\right) + \kern0.5em 0{{.96\mathrm{X}}_1}^2\kern0.5em \left(\mathrm{P}\kern0.5em =\kern0.5em 0.0361\right)\\ {}+\kern0.5em 0{{.70\mathrm{X}}_2}^2\kern0.5em \left(\mathrm{P}\kern0.5em =\kern0.5em 0.0928\right)\ \hbox{-} \kern0.5em 0{{.11\mathrm{X}}_3}^2\kern0.5em \left(\mathrm{P}\kern0.5em =\kern0.5em 0.7630\right)\ \left({\mathrm{R}}^2\kern0.5em =\kern0.5em 0.9540\right)\end{array} $$

### Check point analysis

Two check point batches were prepared and evaluated for the particle size and EE as shown in Table 3. T-test was applied between actual and predicted values of dependent parameters and P-values are reported in Table 3. At 5% significance, there was no significant difference between actual and predicted value of particle size and entrapment efficiency.

**Table 3:** **Checkpoint batches with predicted and measured value**

**Batch code**	**X**_**1**_	**X**_**2**_	**X**_**3**_	**Particle size(nm)**		**% Entrapment efficiency**	
				**Predicted**	**Measure**	**Predicted**	**Measure**
CP1	0	−0.5	0.5	280.54	319 ± 0.4	79.77	77.79 ± 0.2
CP2	0	0.5	0.75	230.27	214 ± 0.8	78.48	80.57 ± 0.17
P-Value				1.00		0.995	

### Optimization of formulation

**Figure 1:**
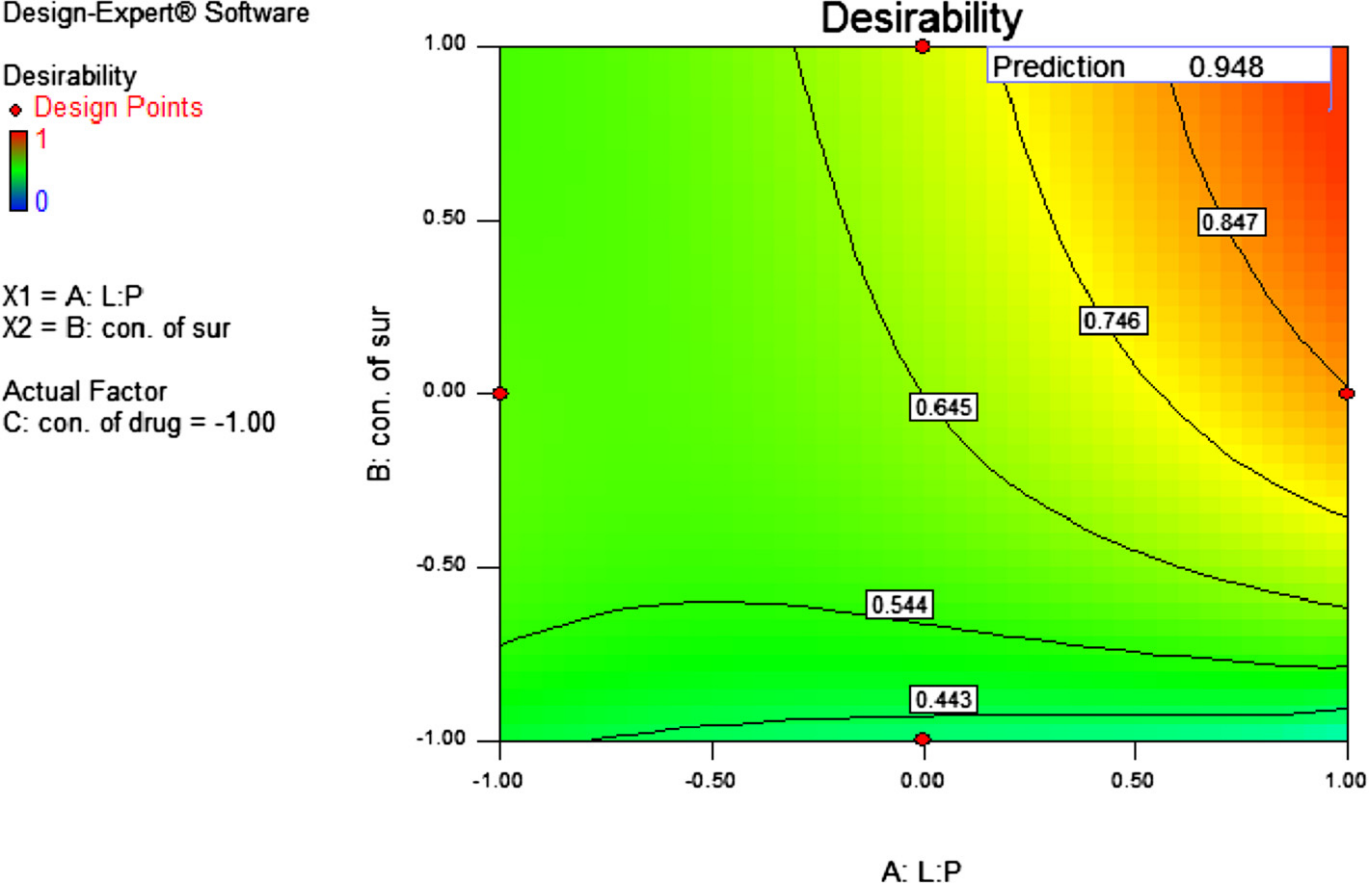
**Desirability plot of the optimize PLHNs formulation.**

After studying the effect of independent variables on the responses, the levels of these variables that give the optimum response were determined. Hence, all the variables were decided in range and the optimum formulation is one that gives lower value of particle size along with a high amount of drug entrapped. Values of the variables for the optimised batch are given in the Table 4. Desirability of the optimized batch was found to be 0.948 which is shown in Figure 1.

**Table 4:** **Optimised formulation as per the design expert® 8.0.6 software**

**Independent variables**	**Criteria**	**Value**	**Desirability**
Lipid: polymer	In range	0.96	0.948
Concentration of surfactant	In range	0.81	
Concentration of drug	In range	−1	
**Dependent variables**			
Particle size	Minimum	228.02	
% Entrapment efficiency	Maximum	83.87

### Particle size, entrapment efficiency and drug loading

Particle size was found to be in the range of 214.0 to 353.0 nm. Particle size of each batch is given in the Table 2. Particle size of the optimized batch was 210.7 ± 1.8 nm and PDI was 0.53 ± 0.67. Entrapment efficiency was found to be in the range of 77.4 to 83.4%. EE of each batch is given in the Table 2. Drug loading of the optimized batch determined by both direct and indirect method were 1.67% and 1.72%, respectively.

### Freeze drying

The freeze dried formulation was found to be soft, white, and amorphous in nature. There was no significant increase in particle size observed (at 5% significant level) after freeze drying as compared to freshly prepared formulation.

### FTIR spectroscopy

**Figure 2:**
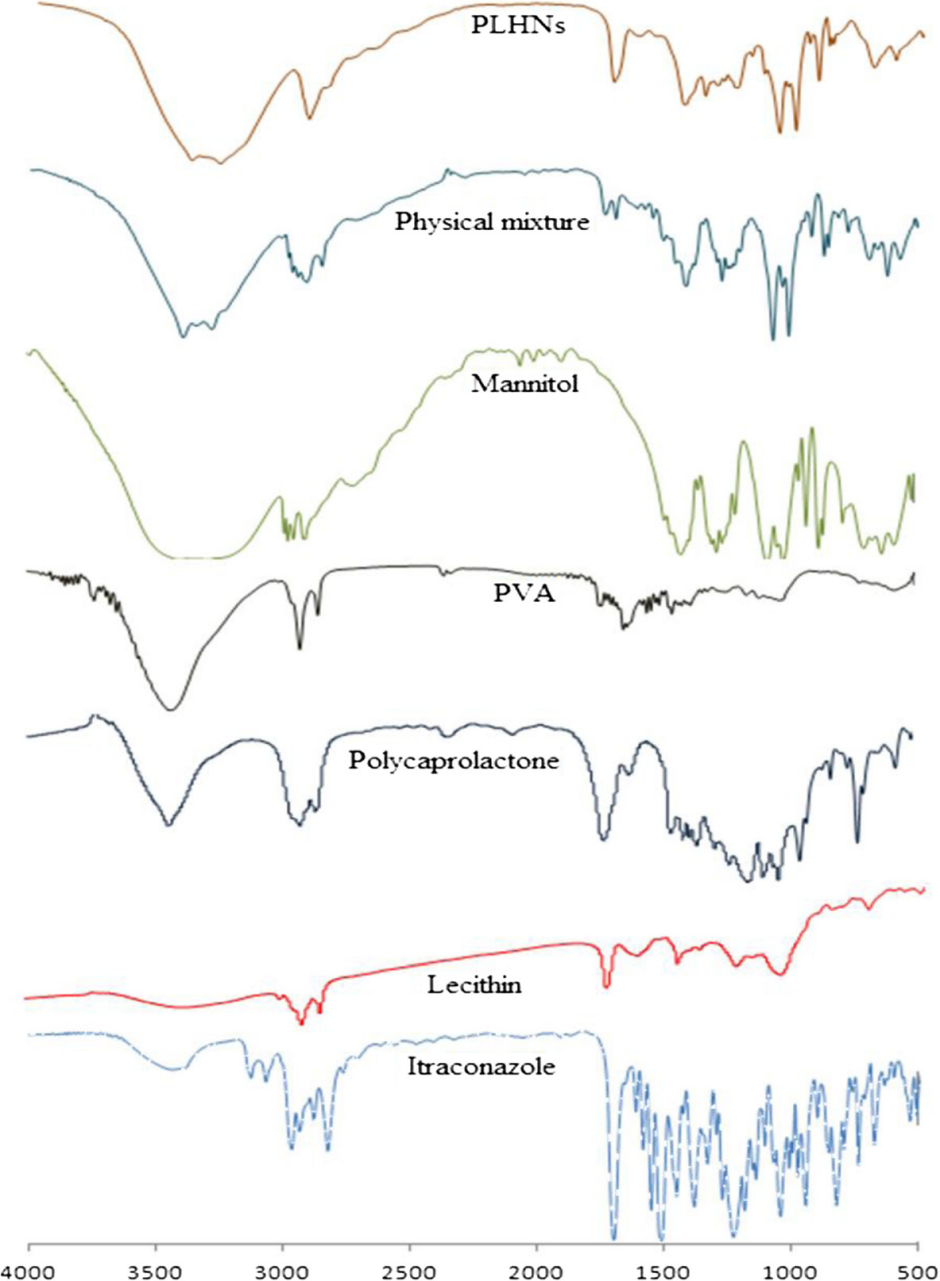
**FTIR spectra of drug, excipients and formulation.**

FTIR spectra of the drug, polymer, lipid, Mannitol, physical mixture and freeze dried formulation are shown in Figure 2. FTIR spectra of freeze dried formulation shows all the characteristic peak of the all components and not shown any additional new peak. The spectra of PLHN shows the characteristic peak of ITZ at 3421.55, 1378.15, 1052.01, 1378.15 cm^−1^ [29], PCL at 2925.28 cm^−1^ and peak of the Soya lecithin at 2925.28, 1437.68 cm^−1^, PVA at 1735.73, 1652.65 cm^−1^ Mannitol at 1247.40 cm^−1^. Thus this result indicates that there was no formation of the new peak, so drug and excipients are compatible with each other and also revealed that ITZ was successfully incorporated into the PLHNs.

### Differential scanning calorimetry (DSC)

**Figure 3:**
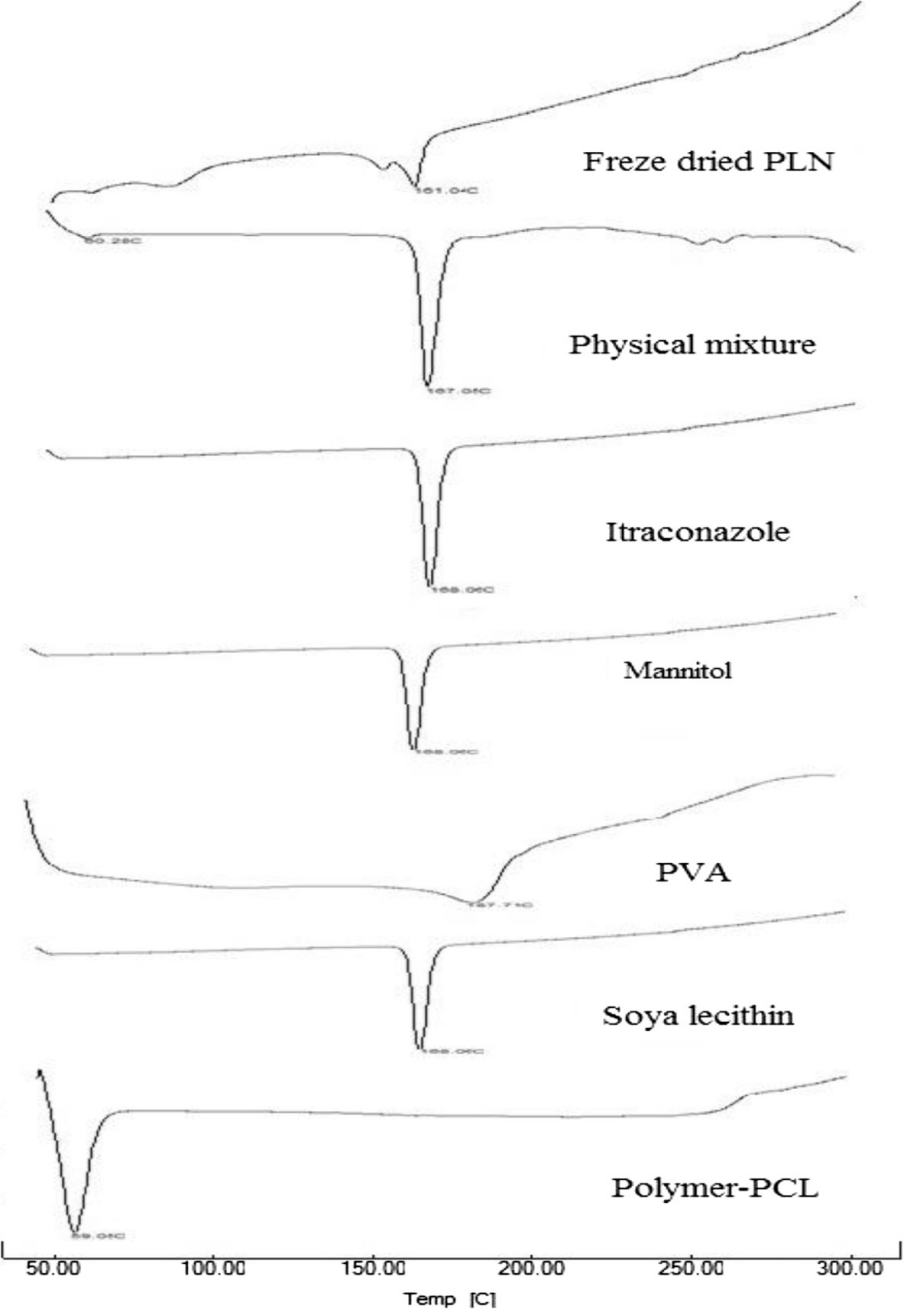
**DSC thermograms of drug, excipients and formulation.**

DSC thermograms of the Formulation, Physical mixture, ITZ, PCL, Soya lecithin, PVA and Mannitol are shown in Figure 3. DSC spectra of formulation and physical mixture gives the sharp peak at 161.0°C and 167.0°C, respectively which is the peak of mannitol and it does not show any new peak or additional peak. So, it is revealed that the excipients are compatible with the drug and there is no any reaction between the drug and excipients [3]. The melting endothermic peak of ITZ was observed at 168.04°C while the thermogram of the lyophilized ITZ incorporated PLHNs does not show the endothermic peak for ITZ [20].

### Powder x-ray diffraction (PXRD)

**Figure 4:**
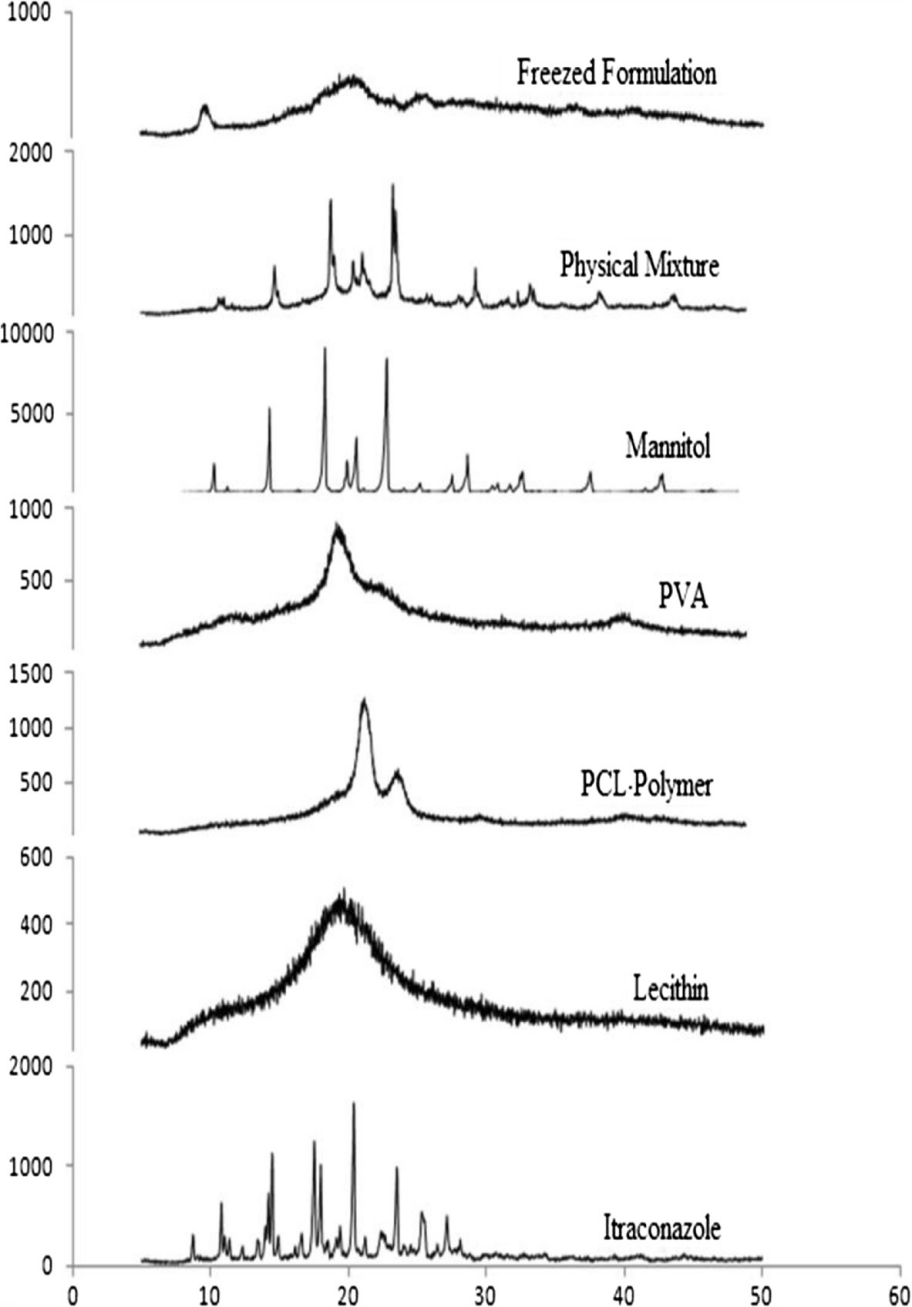
**Powder X-ray diffraction crystallographs of drug, excipients and formulation.**

The diffractograms shown in Figure 4 further confirmed the results of DSC thermal analysis. ITZ powder showed strong typical peaks of crystalline ITZ at 2θ scattered angles 14.49°, 17.53°, 20.38°, 23.5° and 25.29°. The presence of sharp peaks indicates crystalline nature of ITZ. The characteristic peak of ITZ was not observed in PLHNs at corresponding 2θ scattered angles indicating that the drug was encapsulated in the polymer and lipid carrier and also confirms that amorphous form of the formulation [3,20].

### Transmission electron microscopy (TEM) morphology and zeta potential

**Figure 5:**
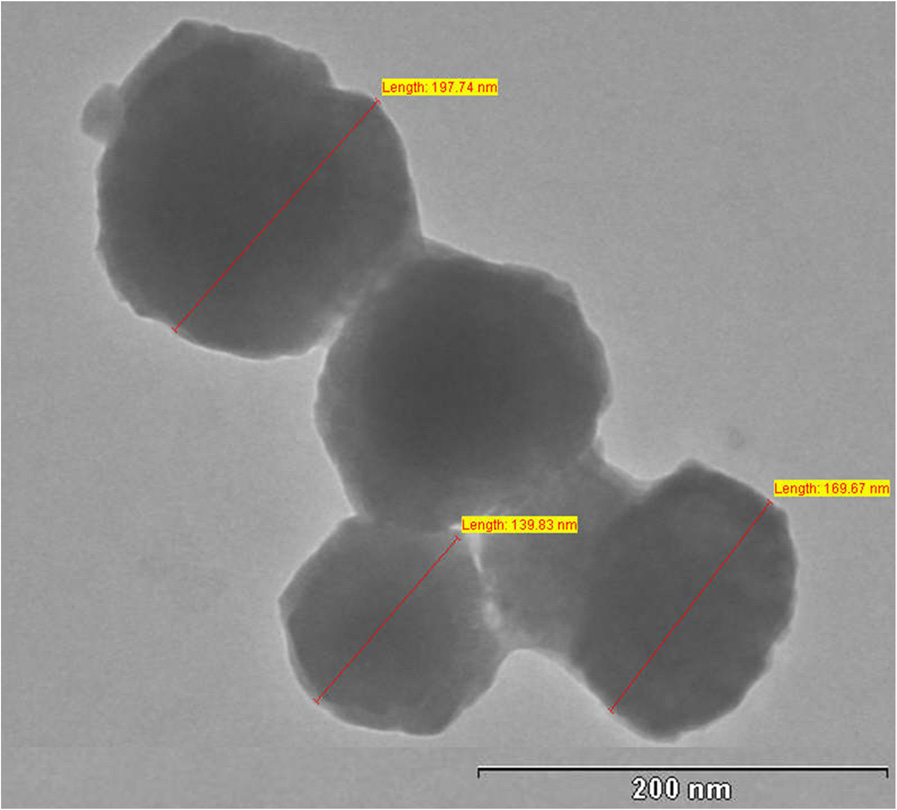
**TEM image of ITZ-PLHNs.**

The TEM image of ITZ-PLHNs is shown in Figure 5. The particles were spherical in shape and show the dim ring of lipid coat surrounding the polymeric core. The particle size was observed between 160–200 nm and is comparable to the results of particle size by particle size analyzer [15]. Zeta potential of the optimized ITZ-PLHNs was found to be −11.7 mV which is attributed to the non-ionic nature of the surfactant PVA as opposed to the anionic nature of the soya lecithin. Because of the negative zeta potential, it produces repulsion between the nanoparticles and prevents the aggregation which gives the long-time stability [5,16].

### In-vitro drug release

With the selection of lipid and polymer ratio, the release kinetics of PLHNs showed some unique features. There was absence of initial burst release observed, may be due to the uniform distribution of ITZ in the PLHNs matrix rather than just on the PLHNs surface. Drug released from the PLHN generally occurs through the drug diffusion and the polymer erosion mechanism. Sustained ITZ release from the PLHNs is attributed to the lipid matrix imparting a barrier to drug release [17].

**Figure 6:**
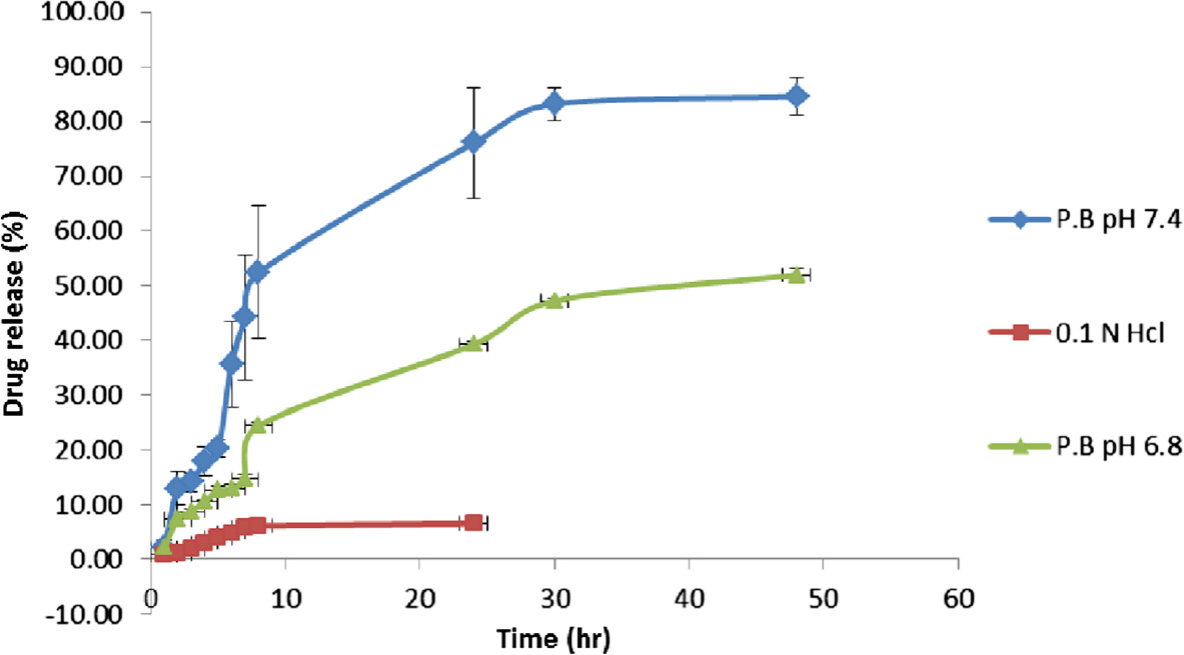
**Drug release profile for ITZ-PLHNs formulation in Phosphate buffer pH 7.4, 0.1 N Hydrochloric acid and Phosphate buffer pH 6.8.**

Drug release profile of the ITZ-PLHNs in phosphate buffer pH 7.4, 0.1 N HCl and phosphate buffer pH 6.8 is shown in the Figure 6. The release profiles were fitted to various kinetic models such as zero-order, first-order, Higuchi equation and Korsmeyer–Peppas equation. ITZ release profile followed Higuchi model in the release media phosphate buffer pH 7.4 ( R^2^ = 0.98) as shown in Table 5 [30,31]. All the kinetic data were fitted to the Korsmeyer-Peppas Equation. Here n > 0.79, the drug is released from polymeric matrix system followed anomalous diffusion mechanism.

**Table 5:** **Kinetic release parameter of ITZ-PLHNs**

**Release media**	**Zero order**		**First order**		**Higuchi**		**Koresmayer-Pepas**	
	**R** ^**2**^	**k**_**0**_ **(h** ^**−1**^ **)**	**R** ^**2**^	**k**_**1**_ **(h** ^**−1**^ **)**	**R** ^**2**^	**k**_**H**_ **(h** ^**-1/2**^ **)**	**R** ^**2**^	**n value**
P.B pH 7.4	0.786	1.77	0.966	−0.01	0.975	15.16	0.9732	0.79

### *Ex-vivo* permeability

**Figure 7:**
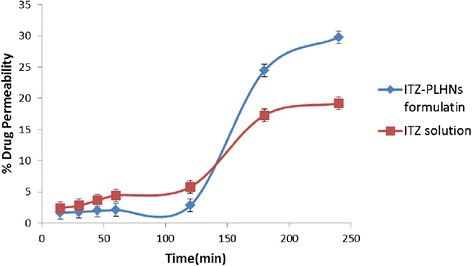
**% cumulative ITZ Permeability of the ITZ solution and formulation.**

*Ex-vivo* permeability studies are relevant approaches to evaluate the absorption enhancing effect of a colloidal drug carrier system on the intestinal tissue. Table 6 shows the comparison of the *ex-vivo* permeability of ITZ solution and ITZ-PLHN formulation. Figure 7 shows the % permeability of ITZ formulation after 240 min is 30%. Measurement of the apparent permeability coefficient (Papp) and the absorption enhancement ratio of PLHNs indicated that there is an increase in the permeability of ITZ from the ITZ-PLHN formulation [25,32].

**Table 6:** **P**_**app**_ **and permeability enhancement ratio of the ITZ solution and formulation**

**Type of formula**	**P**_**app**_ **(cm/sec)**	**Permeability enhancement ratio**
PLHNs Formulation	2.39 × 10^−3^	1.476
ITZ Solution	1.61 × 10^−3^	1

### *In-vitro* cellular uptake study with CLSM

**Figure 8:**
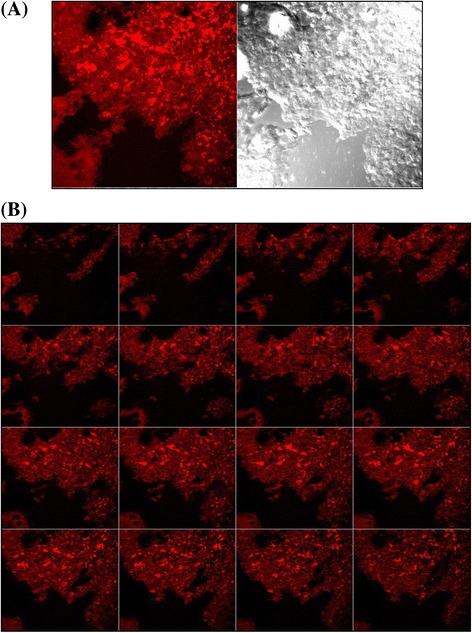
**CLSM images of Rhodamine B labelled ITZ PLHNs A. combined three dimensional images, B. images of intestinal villi optically sectioned in the x-y plane at regularly spaced distances along the z-axis.**

The cellular uptake of the PLHNs was examined to demonstrate the penetration of the nanoparticles across the intestinal barrier and also to study the mechanism of increase in the intestinal permeability of ITZ. The internalization of Rhodamine B loaded NPs incubated for 1 hr was visualized by CLSM. Figure 8(B) shows CLSM images of Rhodamine B labelled PLHNs treated intestinal villi optically sectioned in the x-y plane at regularly spaced distances along the z-axis whereas Figure 8(A) shows combined image containing Rhodamine labelled PLHNs internalized and distributed in the small intestinal mucosal cross-sections. The CLSM images show strong red fluorescent spherical particles in the intestinal villi of rat both on the surface of the intestinal enterocyte and on the M- cells [22]. Villi and microvillus of the intestinal tissue was stained red with the dye and Rhodamine B labelled nanoparticles are highlighted with red fluorescence [28].

### Stability study

**Figure 9:**
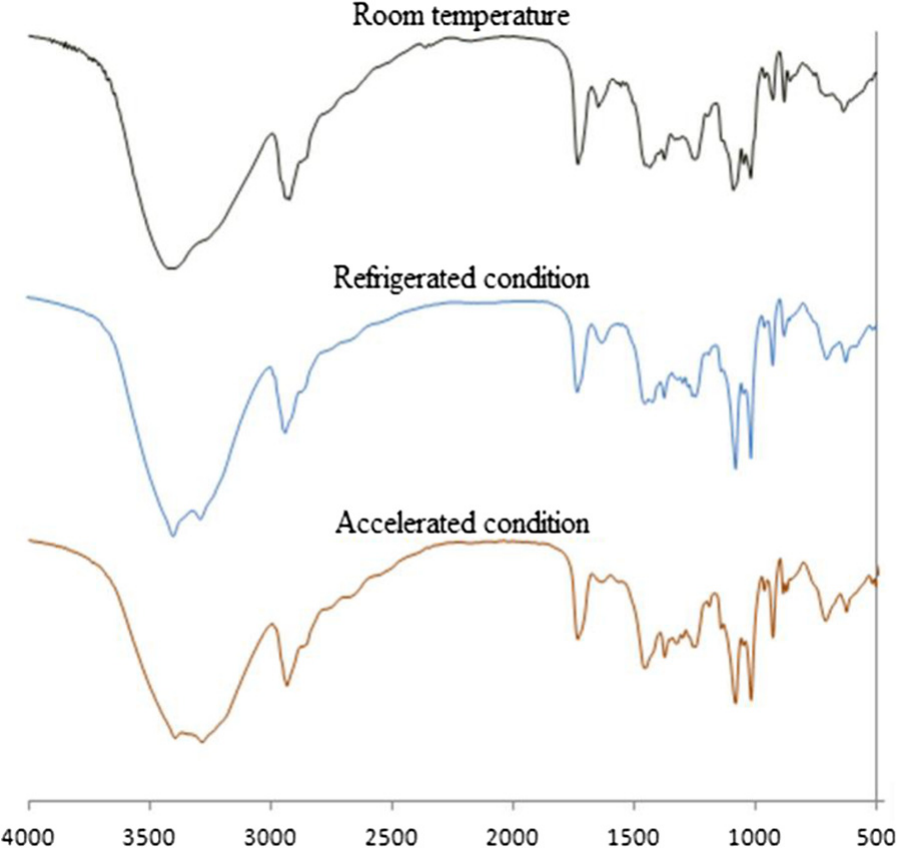
**FTIR spectra of formulation after 45 days for Room temperature, refrigerated condition and accelerated condition.**

Stability study was carried out to know the chemical changes that may occur in the formulations. Table 7 shows the stability study data of particle size and drug content of ITZ-PLHNs formulation. Particle size of the formulation was increased slightly in accelerated, room temperature and refrigeration condition and drug content was decreased slightly from 96% to 95% for all the condition. No significant change in the particle size and drug content was revealed. FTIR spectra of the formulation after 45 days at room temperature, accelerated condition and refrigerated condition are shown in the Figure 9. It was revealed that there is no change as it shows the main peak of the drug intact at 2934.76, 1459.52, 1376.81, 1193.64 cm^−1^.

**Table 7:** **Stability study data for ITZ-PLHNs formulation**

**Day**	**Particle size (nm)**	**Assay (%)**
First	232.8 ± 0.21	96.38 ± 0.02
Accelerated condition		
15	245.3 ± 0.35	96.29 ± 0.04
30	249.5 ± 0.61	95.50 ± 0.03
45	249.9 ± 0.32	95.39 ± 0.02
Room condition		
15	230.3 ± 0.31	96.30 ± 0.04
30	230.8 ± 0.34	96.28 ± 0.05
45	231.4 ± 0.41	96.25 ± 0.021
Refrigerator condition		
15	248.6 ± 0.32	96.12 ± 0.023
30	250.3 ± 0.23	95.43 ± 0.034
45	256.7 ± 0.36	95.28 ± 0.04

## Discussion

### Effects of independent variables on particle size

For the particle size value of the correlation coefficient (R^2^) of the polynomial equation (Equation 4) was found to be 0.9869, indicating good fit of the model. Among the independent variable selected, X_1_, X_2_, X_2_^2^; lipid to polymer ratio, concentration of surfactant and square of the concentration of surfactant, respectively, are significant model terms (P < 0.05).

Here, variable X_1_ and X_2_^2^ have positive effect on particle size as revealed by positive value of coefficient in the equation, it means that as lipid to polymer ratio (X_1_) increases, particle size increases and X_2_ has negative effect on particle size as revealed by negative value of coefficient in the Equation 4 it means that as the concentration of surfactant (X_2_) increases particle size decreases.

**Figure 10:**
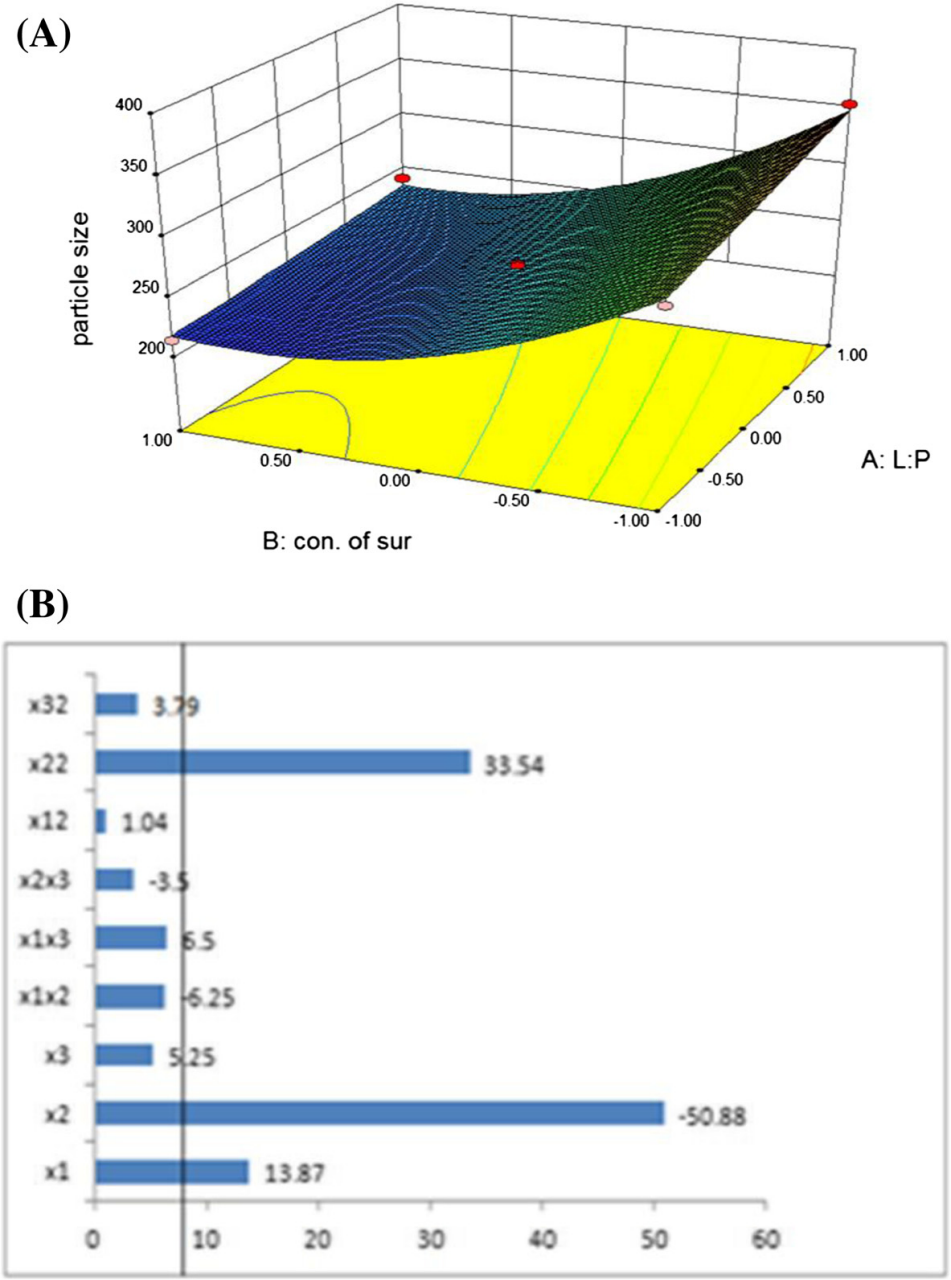
**Response surface 3D plot (A) showing the effect of different variables on particle size and Pareto chart (B) showing the variables having P value greater than 0.05.**

From the response surface 3D plot for the particle size (Figure 10A), it was observed that as the concentration of surfactant increases, particle size decreases. This may be due to the reason that at lower concentration of PVA, it exists as a single molecule layer at surface of particle and at higher concentration it exists as an aggregated form and has an enhanced surfactant activity. It may also be due to effective reduction in the interfacial tension between aqueous and organic phase [33]. As the lipid to polymer ratio increases, particle size increases. This increase in the particle size may be because of increase in the viscosity of the inner polymeric phase that affects the shearing capacity of the mechanical stirrer [15,34].

Thus, the effect of the lipid to polymer ratio and the concentration of surfactant were significant, as it is evident from their high coefficients and the fact that the bars corresponding to variables X_1_, X_2_ and X_2_^2^ extend beyond the reference line in Pareto chart (Figure 10B). P value greater than 0.05 indicates that model terms were not significant so they were removed from the equation to generate the reduced model Equation 6 [19].

6 $$ \mathrm{Reduced}\ \mathrm{model}\ \mathrm{equation}\ \mathrm{f}\mathrm{o}\mathrm{r}\ \mathrm{particle}\ \mathrm{size}\ \left({\mathrm{Y}}_1\right) = + 242.67 + 13.87{\mathrm{X}}_1\hbox{-}\ 50.88{\mathrm{X}}_2 + 33{{.54\mathrm{X}}_2}^2 $$

### Effects of independent variables on entrapment efficiency (EE)

For Entrapment efficiency, the value of the correlation coefficient (R^2^) of the Equation 5 was found to be 0.9540, indicating good fit of the model. Among all the independent variables X_1_, X_2_, X_3_, X_1_^2^; lipid to polymer ratio, concentration of surfactant, concentration of the drug and square of the concentration of drug, respectively, are significant model terms (P < 0.05).

Here, variables X_1_ has positive effect on EE as revealed by the positive value of coefficient in the Equation 5, means as lipid to polymer ratio increases, EE increases and X_2_ and X_3_ has negative effect on EE as revealed by the negative value of coefficient in the Equation 5, it means that as concentration of surfactant and concentration of the drug increases, EE decreases.

**Figure 11:**
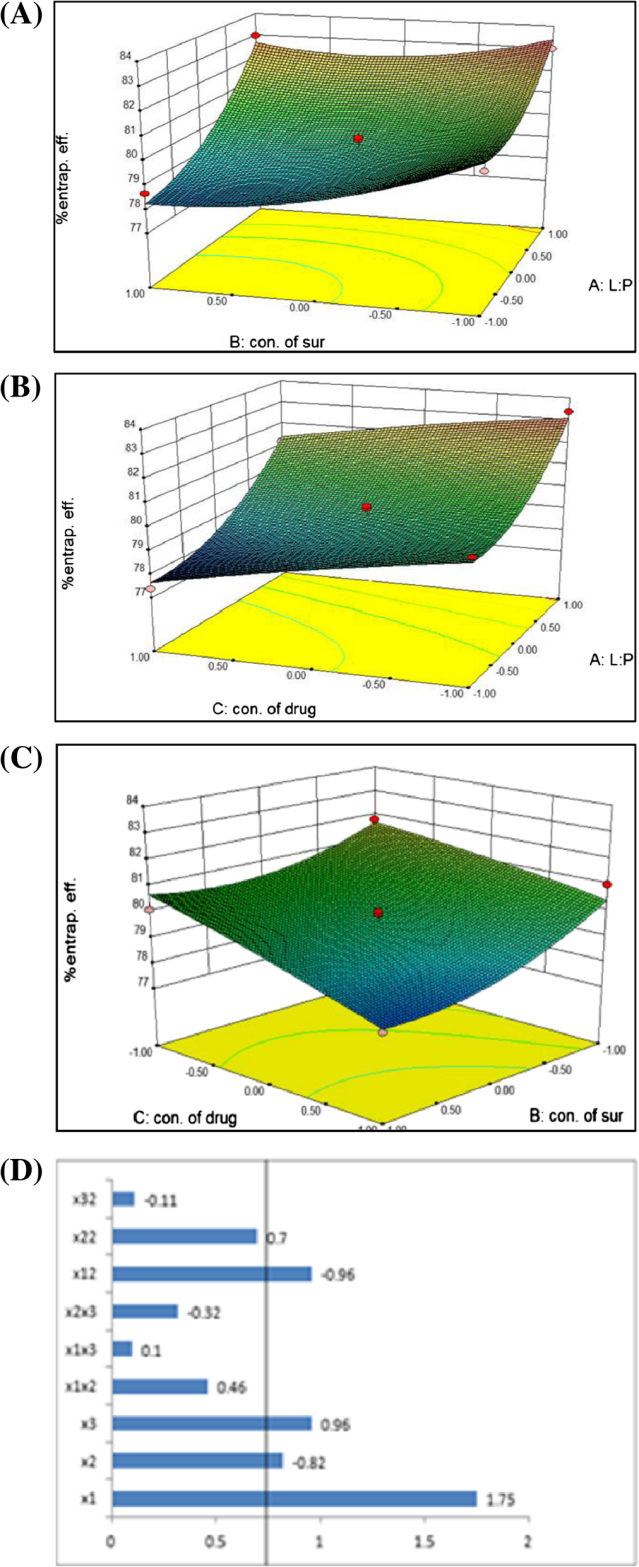
**Response surface graphs (A,B,C) showing effect of different variables on % entrapment efficiency and Pareto chart (D) showing variables having P value greater than 0.05.**

From the response surface 3D plot for the EE (Figure 11A), it shows that as the concentration of surfactant increases, EE decreases. This may be due to decrease in the particle size. It may also be due to increases the partition of the drug from internal to external phase of the medium at the high concentration of surfactant [34]. Figure 11B shows that as the lipid-polymer ratio increases, EE increases. This may be due to increase in the amount of the polymer provides more space to incorporate the drug and lipid layer at the surface of the polymer matrix and also reduces the escaping of the drug into external phase. Figure 11C shows that as the concentration of the drug increases, EE decreases. It may be due to reason that because ITZ is insoluble in water so at high concentration some amount of the drug may precipitate out.

Thus, the effect of the lipid to polymer ratio, the concentration of surfactant and concentration of drug were significant, as it is evident from their high coefficients and the fact that the bars corresponding to variables X_1_, X_2_, X_3_ and X_1_^2^ extend beyond the reference line in Pareto chart (Figure 11D) for the EE. P value greater than 0.05 indicates that the model terms were not significant so they were removed from the equation to generate the reduced model Equation as:

7 $$ \mathrm{Entrapment}\ \mathrm{efficiency}\ \left({\mathrm{Y}}_2\right) = + 79.63 + 1.75{\mathrm{X}}_1\hbox{-}\ 0.82{\mathrm{X}}_2\hbox{-}\ 0.96{\mathrm{X}}_3 + 0{{.96\mathrm{X}}_1}^2 $$

In this study, the model was checked for lack of fit for both the responses; Particle size and EE. For lack of fit P values obtained for particle size and EE were 0.0524 and 0.3965, respectively and hence the current model provided a satisfactory fit to the data (P > 0.05) and has no lack of fit [19].

The derived polynomial equations and contour plots from the Box Behnken experimental design aid in predicting the values of selected independent variables for preparation of optimized PLHN formulations with desired properties. Factorial design was validated by check point analysis. From the result of the check point analysis P value calculated was greater than 0.05 so the model was validated. Optimized batch was selected based on overall desirability factor and having less particle size and high entrapment efficiency.

Optimized formulation was freeze dried to white, amorphous powder which was readily redispersed in to the water. FTIR and DSC of the freeze dried formulation indicate that the drug was satisfactorily incorporated in to the nanoparticles. XRD study revealed that ITZ loaded PLHNs were amorphous in nature. Morphology of PLHNs indicates that it has lipid surrounding the polymeric core and particles were spherical in shape. Optimized formulation was followed the Higuchi model for the drug release which indicates diffusion type of drug release from the matrix. Ex vivo permeability study indicates higher apparent permeability coefficient (Papp) for the ITZ-PLHNs formulation in comparison to the drug solution which confirms increase in drug permeability. This is also indicated by high permeability enhancement ratio.

The NPs absorption occurs in rat follicular mucosa (Peyer’s patches) as well as non-follicular mucosa (normal enterocyte) as visualized in CLSM images. Interaction of NPs with M-cells of the Peyer’s patches would suggest that NPs were concentrated on the follicle associated epithelium promoting the absorption through M cells. The red coloured particles clearly show internalization of the ITZ loaded PLHNs in the intestinal villi. From the results it could be concluded that no single mechanism appears dominant in ITZ loaded PLHNs uptake. Transcellular, Paracellular transport and endocytosis through M-cells of Peyer’s patches may be the mechanisms by which the PLHNs facilitate ITZ absorption.

Stability study of the final optimized formulation revealed that there is no any major change in the particle size and drug content during the time period of 45 days. FTIR spectra of the formulation after 45 days revealed that the drug was in the stable form as the main peak of the drug was present unchanged into the spectra. Thus, Formulation does not give any physical and chemical changes at various environmental conditions for the period of 45 days.

## Conclusion

In the present work, ITZ-PLHNs consisting of the polymeric core and lipid layer at the interface of the core were easily prepared by single emulsification evaporation method with tunable particle size and high entrapment efficiency. Box Behnken design was successfully applied to optimize the effect of lipid to polymer ratio, concentration of surfactant and concentration of drug on particle size and EE. The derived polynomial equations and contour plots aid in predicting the values of selected independent variables for preparation of optimum ITZ formulations with desired properties. Thus, PLHNs may help to improve the oral bioavailability as they directly penetrate in to the systemic circulation by lymphatic uptake, M cells of payer’s patch and paracellular pathway that may reduce the effect of food and hepatic first pass metabolism in comparison with the conventional system.
